# Editorial: Exploring zoonoses: therapeutic strategies and drug mechanisms

**DOI:** 10.3389/fcimb.2025.1581340

**Published:** 2025-03-19

**Authors:** Lei Tan, Mingxiao Ma, Zhanbo Zhu, Shen Yang

**Affiliations:** ^1^ Shanghai Veterinary Research Institute, Chinese Academy of Agricultural Sciences, Shanghai, China; ^2^ College of Animal Science and Veterinary Medicine, Jinzhou Medical University, Jinzhou, Liaoning, China; ^3^ College of Animal Science and Technology, Heilongjiang Bayi Agricultural University, Daqing, Heilongjiang, China; ^4^ Cedars Sinai Medical Center, Los Angeles, CA, United States

**Keywords:** antimicrobial resistance, antiviral therapy, biofilm inhibition, drug mechanisms, zoonoses

Zoonotic diseases continue to challenge both human and animal health, particularly as pathogens evolve and antimicrobial resistance intensifies. In this Research Topic, we have assembled a collection of studies that exemplify innovative therapeutic strategies and deepen our understanding of drug mechanisms against zoonotic pathogens. The articles presented here underscore the potential of natural compounds, novel antiviral concepts, and mechanistic insights to inform future therapeutic development.

## Innovative antibacterial strategies

The study by Zhang et al. explores the antibacterial and antibiofilm activities of a combined formulation of star anise and cinnamon essential oils against multidrug-resistant *Salmonella Thompson*. Isolated from *Bellamya quadrata*, a freshwater snail commonly consumed in Guangxi, these multidrug-resistant strains pose a significant threat to food safety. By demonstrating that the synergistic interaction of these natural compounds effectively inhibits both bacterial growth and biofilm formation, the work offers a promising alternative to conventional antibiotics. This approach not only addresses the critical issue of drug resistance but also highlights the potential of leveraging natural products in antimicrobial therapy.

## Emerging antiviral concepts

Complementing the antibacterial strategy, Maryanchik et al. provide a comprehensive review on antivirotics based on defective interfering particles (DIPs). DIPs are naturally occurring viral variants that interfere with the replication of their parent viruses. This review meticulously discusses the biology of DIPs, their emerging applications as antiviral agents, and the challenges associated with their therapeutic use. The insights presented lay a solid foundation for considering DIPs as a novel class of antivirotics—one that could potentially revolutionize our approach to managing viral infections while minimizing harm to the host.

## Mechanistic insights into antiviral activity

In another original research contribution, Zhao et al. investigate the antiviral effects of melatonin against bovine viral diarrhea virus (BVDV) using MDBK cells. Traditionally recognized for its role in regulating circadian rhythms, melatonin is shown here to significantly inhibit viral replication. The study elucidates that melatonin achieves this effect by modulating endoplasmic reticulum stress and downregulating the NF-κB signaling pathway, in conjunction with the regulation of autophagy. These findings not only broaden the therapeutic profile of melatonin but also provide a mechanistic basis for its potential application in managing BVDV, a pathogen with considerable veterinary and economic implications.

## Exploring plant-derived antivirals

Adding further depth to our topic, Liao et al. present an investigation into the antiviral potential of rosmarinic acid against chikungunya virus (CHIKV). Employing network pharmacology, molecular docking, and *in vitro* experiments, this study demonstrates that rosmarinic acid can effectively suppress CHIKV infection. The results reveal that modulation of the IL-17 signaling pathway plays a key role in mediating the antiviral effects of rosmarinic acid. This work not only validates the antiviral efficacy of a plant-derived compound but also underscores the importance of integrating computational predictions with experimental validations in the drug discovery process.

## Broader implications and future directions

Collectively, the contributions in this Research Topic showcase diverse strategies in combating zoonotic pathogens—ranging from the use of natural compounds to innovative antiviral paradigms. They emphasize the importance of a multidisciplinary approach that bridges natural product research, molecular biology, and pharmacology. As multidrug-resistant bacteria and emerging viruses continue to challenge current therapeutic modalities, the development of alternative strategies is imperative.

Future research should build upon these promising findings by further elucidating the molecular mechanisms underlying the observed therapeutic effects. For instance, deeper exploration into the synergistic interactions of natural compounds may refine their clinical application, while continued investigation into the use of DIPs and the detailed pathways modulated by melatonin and rosmarinic acid could lead to novel antiviral interventions. Ultimately, these studies highlight the value of integrating traditional medicine with modern molecular techniques—a synergy that holds significant promise for future drug development.

## Concluding remarks

The articles featured in this Research Topic not only advance our understanding of therapeutic strategies against zoonotic diseases but also pave the way for innovative approaches in both human and veterinary medicine. I extend my sincere gratitude to all contributing authors and the dedicated reviewers whose efforts have been invaluable in shaping this Research Topic. It is my hope that the insights and discoveries presented here will stimulate further interdisciplinary collaboration and drive the next wave of innovations in the treatment and prevention of zoonoses.

